# Enhanced renoprotective effect of HIF-1α modified human adipose-derived stem cells on cisplatin-induced acute kidney injury *in vivo*

**DOI:** 10.1038/srep10851

**Published:** 2015-06-05

**Authors:** Wei-Wei Wang, Ze-Zheng Li, Wei Wang, Yan Jiang, Jin Cheng, Shi Lu, Jin-Yuan Zhang

**Affiliations:** 1 Nephrology Division, Jimin Hospital, Shanghai 200052, China

## Abstract

Current therapeutic options for acute kidney injury (AKI) are limited to the use of supportive measures and dialysis. A recent approach that has sparked great interest and gained enormous popularity is the implantation of stem cells to repair acutely damaged kidney organ. Hypoxia inducible factor-1α (HIF-1α) is effective in protecting the kidney from ischemia and nephrotoxicity. In this study, we investigated whether HIF-1α-modified adipose-derived stem cells (ASCs) had an enhanced protective effect on cisplatin-induced kidney injury *in vivo*. Cisplatin-induced AKI was established in nude mice. Our study demonstrated that HIF-1α-modified ASCs obviously promoted the recovery of renal function, ameliorated the extent of histologic injury and reduced renal apoptosis and inflammation, but HIF-1α-modified ASCs homed to kidney tissues at very low levels after transplantation. In addition, we also found that HIF-1α-modified ASCs significantly increased HO-1 expression in cisplatin-induced AKI *in vivo*. Thus, our study indicated HIF-1α-modified ASCs implantation could provide advanced benefits in the protection again AKI, which will contribute to developing a new therapeutic strategy for the treatment of AKI.

Although recent advances have improved our understanding of acute kidney injury (AKI), AKI remains a high risk factor for mortality and morbidity. Since 2008, multiple observational studies have shown a substantial proportion of patients with AKI, even those without previous kidney disease, often recover some degree of renal function but then have progression to advanced stages of chronic kidney disease^1^. Current therapeutic options for AKI are limited to the use of supportive measures and dialysis. Furthermore, several therapeutic agents used in clinical practice have been reported to produce functional impairment and kidney injury^2^, and supportive measures require patients to wait for renal function to recover^3^,^4^. Therefore, it is imperative to accelerate the development of new and more effective strategies for the treatment of AKI.

An approach that has sparked great interest and gained enormous popularity is the utilization of stem cells to repair acutely damaged organs. Over the past decade, efforts have been made to explore the use of bone marrow-derived mesenchymal stem cells (BM-MSCs) in the treatment of renal injury and disease. Data suggest that BM-MSCs may prevent the progression of renal disease or facilitate the repair of damaged renal tissue through the production of secreted factors or the differentiation of stem cells into cells that can re-populate the damaged tissue^5^,^6^. Recent studies have suggested that adipose tissue is an attractive source of multipotent stem cells, which possess the ability to differentiate into osteogenic, chondrogenic, myogenic, neurogenic or endothelial cells^7^,^8^,^9^,^10^. Additionally, ASCs show a high proliferation rate and low senescence rate even when harvested from adults, and they do not trigger immune rejection^11^. Thus, it seems that adipose tissue is a promising tissue source for stem cells, with powerful implications for regenerative medicine.

Hypoxia-inducible factor (HIF), a basic helix-loop-helix transcription factor composed of alpha (α) and beta (β) subunits, is a master regulator that mediates the adaptive response to hypoxia in cells and tissues. HIF-β is stable and can be detected in the cytoplasm and nucleus under both normoxic and hypoxic conditions. Under hypoxic conditions, HIF-α protein accumulates and is transported into the nucleus, where it dimerizes with HIF-β, initiating the transcription of HIF target genes^12^. HIF-α is rapidly degraded because it contains an oxygen-dependent degradation (ODD) domain. Elson and colleagues reported that cells transfected with cDNA encoding HIF-1α in which the ODD was deleted showed constitutively active HIF-1α signaling regardless of oxygen tension and that deletion of the ODD domain (ΔODD) did not affect the function of HIF-1α^13^, a finding which may be beneficial in exploring the effect of HIF-1α on damaged tissue under normoxic conditions.

Researchers have demonstrated that the activation of HIF, especially HIF-1α, is effective in treating various kidney diseases including ischemia or nephrotoxic AKI^14^,^15^. Other reports have also demonstrated that HIF-1α may activate the expression of downstream renal-protective genes, such as heme oxygenase-1 (HO-1), which is effective in protecting kidney from the damage^16^,^17^. Based on these findings, we investigated the enhanced protective effect of lentivirus-mediated HIF-1α (ΔODD) overexpression in hASCs against cisplatin-induced nephrotoxicity *in vivo.*

## Result

### Protection of HIF-1α-hASCs on renal function and histology of cisplatin-induced AKI

**Figure 1:**
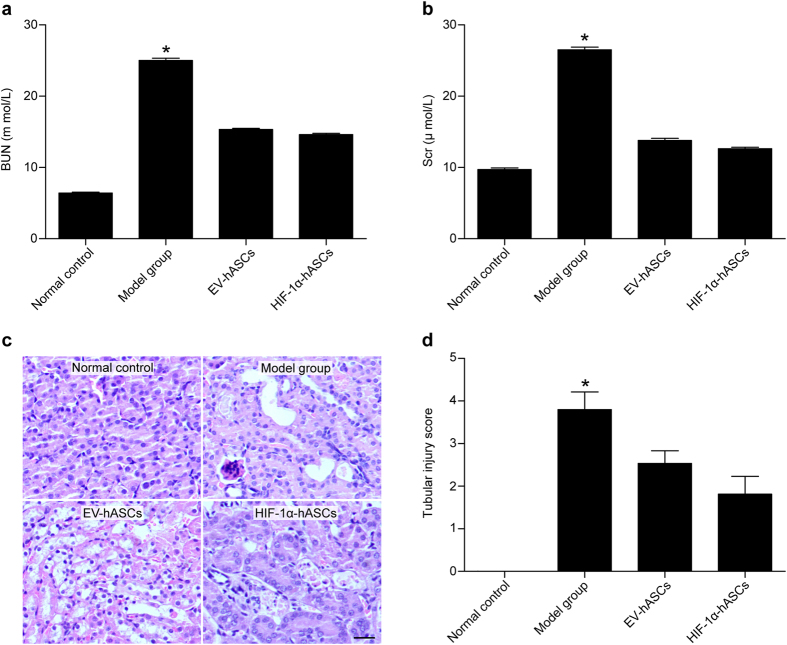
Renal function and histopathological analysis. (**a**) The change of blood urea nitrogen (BUN). (**b**) The level of serum creatinine (Scr). (**c**) H&E staining of kidney tissues. (**d**) Tubular damage scores. * *P* < 0.05 compared with other groups. Scale bar: 100 μm.

EV-hASCs and HIF-1α-hASCs exhibited an obviously renoprotective effect, as reflected by lower BUN and SCr levels compared with the model group (*P* < 0.05) (Fig. 1 A, B). EV-hASCs and HIF-1α-hASCs also relieved renal morphological injury, as indicated by fewer necrotic tubules and casts compared with the model group (Fig. 1C). In addition, the tubular damage scores were decreased significantly in EV-hASCs and HIF-1α-hASCs groups compared with those in the model group (*P* < 0.05) and which was much lower in HIF-1α-hASCs group (Fig. 1D).

### Effect of HIF-1α-hASCs in reducing renal cell apoptosis of cisplatin-induced AKI

**Figure 2:**
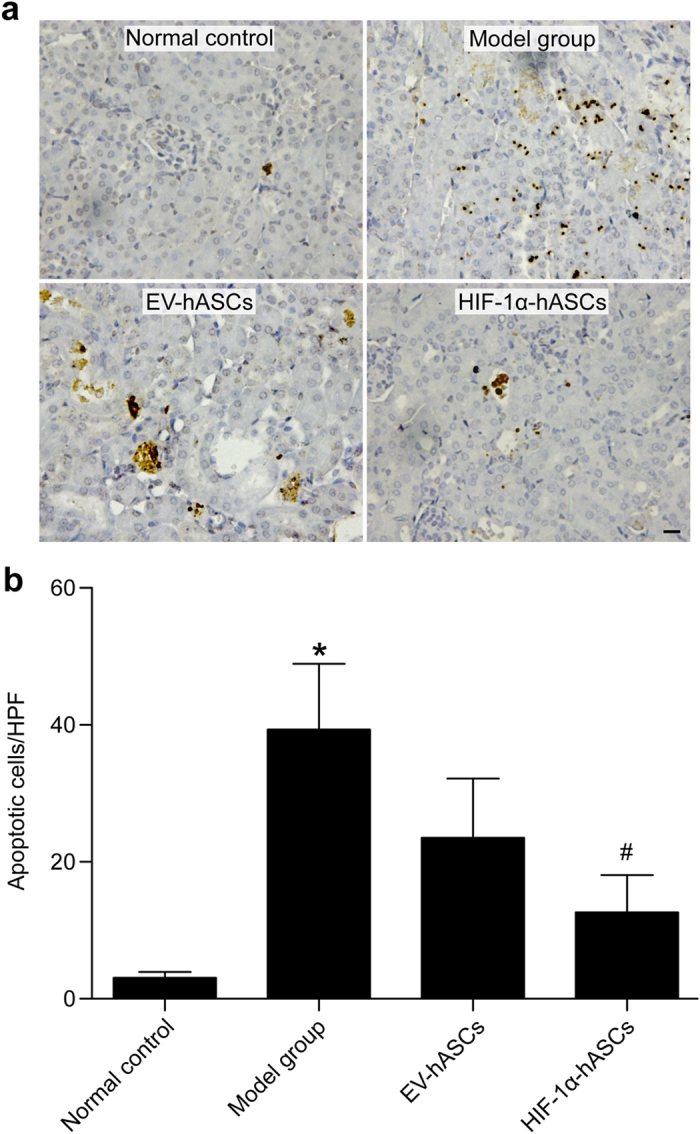
Evaluation of cellular apoptosis in kidney tissues. (**a**) TUNEL staining. (**b**) Quantification of apoptotic cells. * *P* < 0.05 compared with other groups, # *P* < 0.05 compared with EV-hASCs group. Scale bar: 100 μm.

To analyze cisplatin-induced apoptosis, kidney sections were examined after detecting DNA fragmentations with the *in situ* TUNEL assay. The brown nuclei stained by the TUNEL assay were easily observed in the model group, yet the stained nuclei were decreased in the EV-hASCs and HIF-1α-hASCs groups (Fig. 2A). To further evaluate the antiapoptotic activity of EV-hASCs and HIF-1α-hASCs, the apoptotic indexes were calculated. The number of TUNEL-positive cells in the tubular epithelium was significantly increased in cisplatin-treated nude rats, and the apoptotic indexes were significantly reduced in nude rats that had received an infusion of EV-hASCs and HIF-1α-hASCs (*P* < 0.05 vs. model group), and the score of the apoptotic indexes was lowest in the HIF-1α-hASCs group (*P* < 0.05 vs. EV-hASCs; Fig. 2B).

### Regulation of HIF-1α-hASCs on inflammatory cytokines level of cisplatin-induced AKI

**Figure 3:**
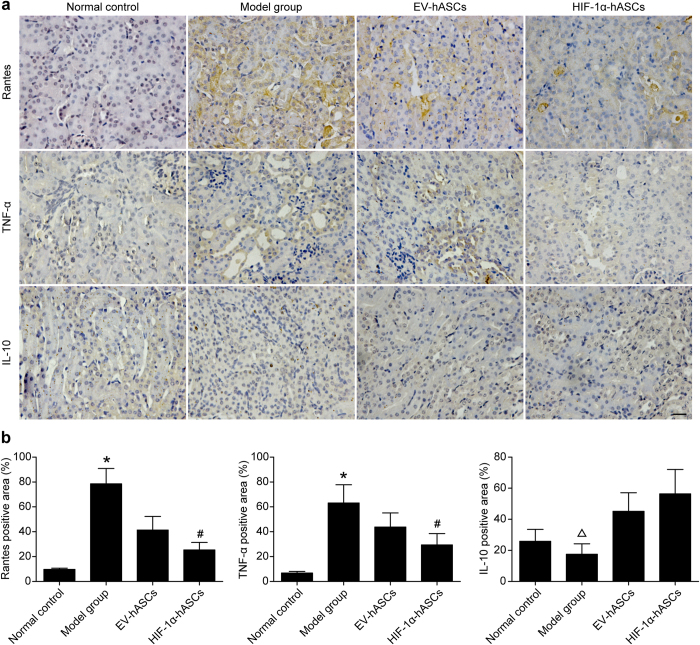
Expression of inflammatory cytokines in kidney tissues. (**a**) Immunohistochemical staining of RANTES, TNF-α and IL-10 in renal tissues. (**b**) Histological scores. * *P* < 0.05 compared with other groups, # *P* < 0.05 compared with EV-hASCs group, Δ *P* < 0.05 compared with EV-hASCs and HIF-1α-hASCs groups, Scale bar: 100 μm.

To investigate the effect of HIF-1α-hASCs on regulating inflammatory cytokines level, the immunohistology of RANTES, TNF-α and IL-10 was performed in renal tissue of mice suffering from cisplatin-induced AKI. The immunohistological area percentage of positive staining indicated that the expression of pro-inflammatory cytokines (RANTES and TNF-α) was significantly increased in cisplatin-treated nude rats (*P* < 0.05 vs. other groups), and HIF-1α-hASCs obviously decreased the expression of pro-inflammatory cytokines (*P* < 0.05 vs. EV-hASCs). Cisplatin-induced decrease in the anti-inflammatory cytokines expression (IL-10) in the kidney was significantly increased by EV-hASCs and HIF-1α-hASCs treatment (*P* < 0.05 vs. EV-hASCs and HIF-1α-hASCs; Fig. 3).

### Existence and differentiation of hASCs in cisplatin-induced AKI

**Figure 4:**
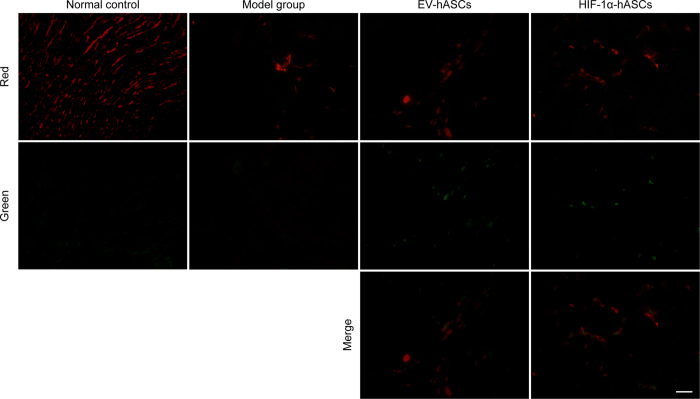
Distribution of GFP-labeled-hASCs in renal tissues under fluorescence microscope. Scale bar: 200 μm.

To evaluate the migration and differentiation of hASCs, GFP-positive hASCs and Cy3-labeled CK-18 positive renal tubular cells were observed in the renal tissue. GFP-positive hASCs were detected in EV-hASCs and HIF-1α-hASCs but the number of cells was rare. CK-18, renal tubular cells marker, was uniformly showed in the control group, and the expression of CK-18 was obviously decreased in cisplatin-induced renal tissue. The pictures of GFP-positive hASCs and Cy3-labeled CK-18 positive renal tubular cells were merged in EV-hASCs and HIF-1α-hASCs and their results showed that rare cells were overlapped, which showing rare differentiation of hASCs into renal tubular cells (Fig. 4).

### Expression of HIF-1α-regulated HO-1 gene in cisplatin-induced AKI

**Figure 5:**
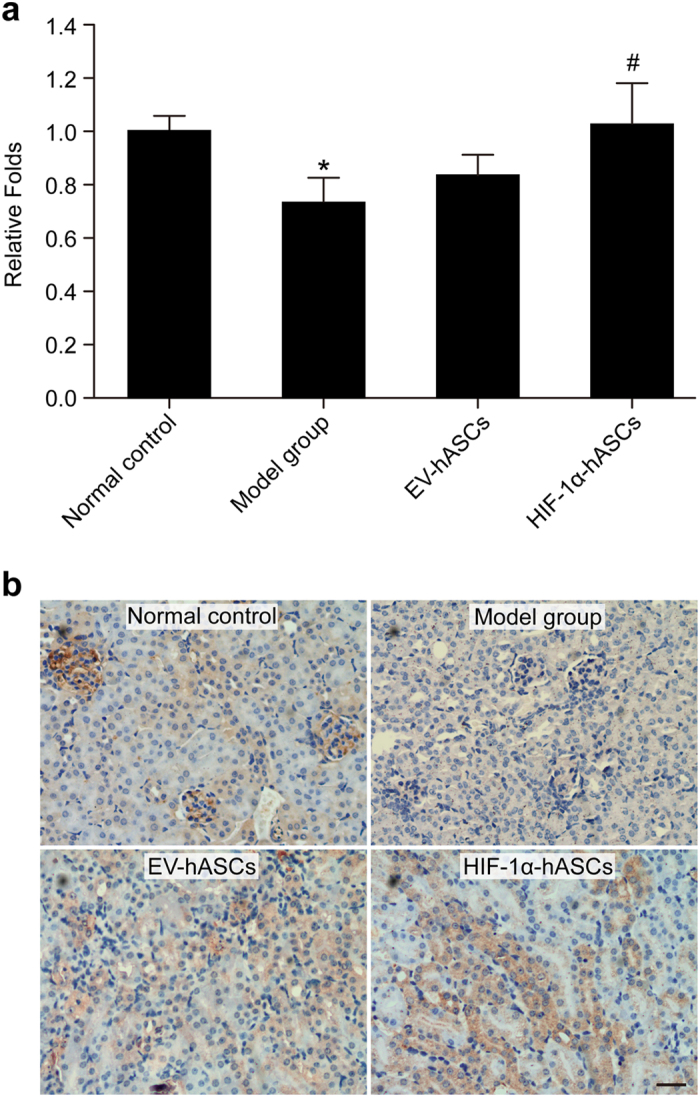
Analysis of HO-1 expression. (**a**) The expression of HO-1mRNA was tested by RT-PCR. * *P* < 0.05 compared with the model group, # *P* < 0.05 compared with EV-hASCs group. (**b**) The expression of HO-1 protein was examined by immunohistology. Scale bar: 200 μm.

To test whether paracrine effect on renal function in cisplatin-treated rats, the expression of HIF-1α-downstream renal protective-gene (HO-1) was performed by RT-PCR. The mRNA expression of HO-1 was significantly decreased in the model group (*P* < 0.05 vs. control group). The mRNA expression of HO-1 was obviously increased in EV-hASCs and HIF-1α-hASCs, compared to the model group (*P* < 0.05), and their expression was also showed significance between HIF-1α-hASCs and the model group (*P* < 0.05). The immunohistological analysis of HO-1 protein was shown to be identical to its mRNA expression (Fig. 5).

## Discussion

AKI is a common result of ischemic or toxic renal injury. A growing body of evidence indicates that cisplatin has direct cytotoxic effects on most cultured cells *in vitro* and tissues *in vivo* and can contribute to the development of AKI through damage to proximal tubules in the kidney. Thus, cisplatin is often used in the study of AKI. The mechanism by which cisplatin achieves its nephrotoxic effect is complex and includes inducing apoptosis, stimulating inflammation and promoting fibrogenesis^18^. In our previous studies, we successfully delivered the HIF-1α gene into hASCs via a lentiviral vector, which increased the levels of HIF-1α expression and maintained their stem cell characteristics *in vitro*^19^. In this study, we observed the effect of HIF-1α-modified hASCs on cisplatin-induced nephrotoxicity *in vivo*. To the best of our knowledge, this report is the first of show that hASCs modified by HIF-1α offered better protective effects on renal function and tubular structure by decreasing renal tubular apoptosis, suppressing inflammation and stimulating HO-1 gene expression, and our study also demonstrated that a paracrine effect played an important role in treating cisplatin-induced AKI in nude rats by the implantation.

In our study, two essential indices of renal function, the serum levels of BUN and creatinine, were substantially increased in cisplatin-induced AKI compared with normal control. Furthermore, histopathological renal injury scores were significantly higher in AKI animals than in normal controls. *In vitro* implantation demonstrated that hASCs modified by HIF-1 α was superior to hASCs alone at improving impaired renal function and lessening the damage of the kidney.

Apoptosis, programmed cell death, occurs as part of normal physiological processes in many multicellular organisms. However, excessive apoptosis can lead to tissue damage and a loss of tissue function. In cisplatin-induced AKI, apoptosis is a major cause of tubular cell loss and attenuation of tubular cell apoptosis leads to amelioration of nephrotoxicity in AKI^20^. So the inhibition of apoptosis may be a powerful therapeutic strategy for the treatment of cisplatin-induced AKI. Our results indicated that hASCs showed an anti-apoptotic effect in AKI, and hASCs modified by HIF-1 α obviously increased the anti-apoptotic effect, as shown by a significant reduction in the number of TUNEL-positive cells.

Inflammation is well recognized to have an important role in cisplatin nephrotoxicity^21^,^22^. Study indicated that alpha-lipoic acid attenuated cisplatin-induced acute kidney injury in mice by suppressing renal inflammation^23^. Other report also clearly showed that treatment with pharmacological inhibitors and antibodies against TNF-α, or genetic targeting of TNF, might blunt the increases in IL-β, transforming growth factor-β, MCP-1 RANTES, and MIP1, being generated by cisplatin nephrotoxicity, which is associated with marked resistance to cisplatin-induced renal injury and tubular cell death^24^. Our study showed that the expression of inflammatory cytokines (RANTES and TNF-α) was remarkably reduced by infusion of hASCs. In addition to the effects on inflammatory cytokines, we also observed increased level of IL-10, the anti-inflammatory cytokine, suggesting the broad targets affected by hASCs. In the present study, of particular importance, was that hASCs modified by HIF-1α provided a significantly more profound effect at reducing inflammatory mediators and increasing anti-inflammatory cytokines.

The functional recovery that occurred following stem cells infusion was considered that it might be achieved through tubular protection and/or enhanced renal function due to paracrine mechanisms or the replacement of damaged cells by differentiated stem cells. Some reports suggested that stem cells, especially mesenchymal stem cells (MSCs), had protective effects against AKI arising from chemical (glycerol and cisplatin) and ischemia-reperfusion (I/R) injuries by secreting beneficial factors such as angiogenic proteins, reparative cytokines and growth factors such as VEGF, HGH and IGF^25^,^26^. Dynamic secretion of these growth factors by MSCs implies that MSCs can aid in the regeneration and repair of injured tissue^27^. Our study showed that the homing of hASCs to sites of kidney injury and integration was rare in models of AKI, and hASCs exerted the therapeutic effects by primarily paracrine actions on the injured kidney by releasing protective factor such as HO-1. In the present study, hASCs modified by HIF-1α showed a more effective therapy by increasing the release of the protective factor.

In conclusion, we found that hASCs had a protective effect on tubular injury *in vivo*, and hASCs modified by HIF-1α could strengthen the renoprotective effect of stem cells by decreasing renal tubular apoptosis, suppressing inflammation and secreting renal-protective factor. So our study indicated that this combined strategy of stem cell transplantation with gene therapy would prove to be an optimized approach for the treatment of renal disease such as AKI, which will contribute to promoting the application of genes modifying stem cells in the clinical.

### Materials and Methods

#### Human adipose-derived stem cells (hASC) culture and preparation

hASCs, purchased from Cyagen Biosciences Inc., were maintained in culture medium including Dulbecco’s Modified Eagle Medium (DMEM), 10 % (v/v) fetal bovine serum, 50 U penicillin ml−1 and 50° μg streptomycin ml−1 at 37 °C/5% CO2. At 80% confluence, cells were trypsinized with 0.25% trypsin/EDTA and passaged into new flasks for further expansion. The medium was changed every other day.

#### Lentivirus production

HIF-1α (ΔODD) was amplified from pcDNA3-HIF-1α (401Δ603; a gift from Dr. Franklin) by PCR using primers containing BamHI and AscI restriction sites. HIF-1α (ΔODD) was constructed into lentivirus-expressing vector containing green fluorescent protein (GFP) according to the Invitrogen protocol. Viral particles were harvested and stored at −80 °C. The empty lentiviral vector was generated using the same procedure. (HIF-1α over-expression refers to HIF-1α (ΔODD) over-expression.)

#### Lentiviral transfection of hASCs

hASCs were transfected according to our previous report^19^. Briefly, hASCs were plated in 25  cm^2^ flasks and grown to 80 % confluence (~10^6^ cells). Cells were incubated overnight with lentivirus at a multiplicity of infection (moi) of 1 in the presence of 8 μg polybrene ml−1 (Sigma, USA), and the medium was replaced with 5 ml fresh medium the next day. Three days later, GFP-expressing cells were collected by FACS and re-plated for further culture.

#### Animal experiments

Care and handling of animals and all experimental procedures used here were approved by Shanghai University of Traditional Chinese Medicine and in accordance with standards established by the National Institutes Health Guide for the Care and Use of Laboratory Animals. For induction of AKI, male-BALB/c nude mice (approximately 18–22 g) were given an intraperitoneal injection of cisplatin (10 mg/kg body wt) on 2 successive days (day 0 and day 1, total dosage 20 mg/kg). Twenty-four hours after the second cisplatin dose (day 2), nude mice were divided into three groups and received intravenous tail injections as follows: Group 1, the model group (saline, 200 μl; n = 6); group 2, lentivirus-mediated empty vector-transfected hASCs (EV-hASCs) (1 × 10^5^ cells/200 μl; n = 6); group 3, lentivirus-mediated HIF-1α-transfected hASCs (HIF-1α-hASCs) (1 × 10^5^ cells/200 μl; n = 6). Normal control animals (n = 5) did not receive cisplatin injections. Nude mice were killed day 5.

Renal function was assessed in terms of serum creatinine (SCr) and blood urea nitrogen (BUN) determined by automatic biochemical analyzer (Roche Diagnostics). Kidneys were harvested for histological analysis.

#### Renal histology

Kidney samples were immersed in 4% neutral buffered formaldehyde, fixed for 24 h, embedded in the paraffin, deparaffinized with xylene and rehydrated in an alcohol series and water, and then sliced into 4 μm sections for histological analysis.

Hematoxylin and eosin (H & E) staining was performed, and the sections were evaluated according to a previously described semiquantitative scale designed to evaluate the degree of tubular necrosis^28^. 0, normal kidney; 1, minimal necrosis (<5% involvement); 2, mild necrosis (5 to 25% involvement); 3, moderate necrosis (25 to 75% involvement); 4, severe necrosis(>75% involvement). The evaluation was recorded in a single-blind manner.

#### Tubular apoptosis

To quantify tubular apoptosis, TUNEL (terminal deoxynucleotidyltransferase -mediated dUTP nick end-labeling) staining was performed using the *in situ* Apoptosis Detection Kit (Boster Biotech Co, China) according to the manufacturer's instructions. Briefly, kidney sections were deparaffinized, rehydrated, digested with proteinase K, and labeled with a TUNEL reaction mixture for 60 min at 37 °C. To reveal the total nuclei, the same slides were stained with hematoxylin. Cells with apoptotic nuclei were counted in at least 10 different fields and were expressed as the percentage of the total cells counted.

#### Immunohistochemistry and Immunofluorescence

Renal inflammatory factors (RANTES, TNF-α and IL-10) and HO-1 were detected by immunohistochemistry. Briefly, paraffin-embedded kidney sections were deparaffinized with xylene and rehydrated in an alcohol series and water. Samples were incubated with anti-human primary antibodies including RANTES (Abcam, USA), TNF-α (Abcam, USA), IL-10 (Abcam, USA) and HO-1 (Abcam, USA) overnight at 4 °C, then rinsed with PBS three times and incubated with biotinylated secondary antibody. Nuclei were visualized by counterstaining with Harris hematoxyline.

To observe *in vivo* distribution of administered ASCs in the kidney, immunofluorescence of cytokeratin 18 (CK18, epithelial cell marker) was performed. Briefly, paraffin-embedded kidney sections were deparaffinized with xylene and rehydrated in an alcohol series and water. Samples were incubated with anti-human primary antibody at 4 °C overnight. Then sections were washed with PBS-T (1 × PBS containing 0.05% Tween 20) and incubated with Cy3-labeled secondary antibody.

#### Real-time PCR

Total RNA was isolated from the cells using TRIZOL and the reverse transcription of the purified RNA was performed using oligo (dT) priming and superscript II reverse transcription, according to the manufacturer’s instruction (Invitrogen, USA). Real time-PCR was performed using SYBR green. The primers’ sequences were as follows: the primers 5’-CTGCTCAACATCCAGCTCTTTG-3’ (forward) and 5’- CAACTGTCGCCACCAGAAAG -3’ (reverse) were designed to amplify HO-1 and the primers 5’- AGGCACCAGGGCGTGAT -3’ (forward) and 5’- GCCCACATAGGAATCCTTCTGAC-3’ (reverse) were designed to amplify β-actin.

#### Statistical analysis

Samples values are expressed as the mean ± standard deviation (S.D.). Data were analyzed by ANOVA using the SPSS13.0 statistical software package. *P*-values less than 0.05 were considered significant.

## Additional Information

**How to cite this article**: Wang, W.-W. *et al.* Enhanced renoprotective effect of HIF-1a modified human adipose-derived stem cells on cisplatin-induced acute kidney injury *in vivo*. *Sci. Rep.* **5**, 10851; doi: 10.1038/srep10851 (2015).
